# Clinician Perspectives on Government Versus Private Psychiatry Services in Bangladesh: A Thematic Analysis

**DOI:** 10.1192/bjo.2026.11600

**Published:** 2026-06-30

**Authors:** Mehtab Ghazi Rahman, Ashik Mahmud, Lisanul Hasan, Ishrat Jahan, Mursalin Mosaddeque

**Affiliations:** 1 Central & North West London NHS Foundation Trust, London, United Kingdom; 2 Neuroscience & Psychiatry Hub Ltd., Dhaka, Bangladesh

## Abstract

**Aims:**

In Bangladesh, mental healthcare is delivered through parallel government and private psychiatric services. Both sectors play a critical role in service provision but there is limited empirical evidence comparing how these systems operate in practice from the perspective of clinicians working across both settings.

This study aimed to explore and compare clinician experiences of working in government and private psychiatric services in Bangladesh. We hypothesised that government services would be characterised by higher patient volume and resource constraints, while private services would offer more structured multidisciplinary care, greater patient autonomy, and improved continuity, albeit with increased administrative demands and financial barriers.

**Methods:**

A qualitative thematic analysis was conducted using structured written responses from 24 multidisciplinary mental health professionals who had worked in both government and private psychiatric settings. Participants included psychiatrists, resident medical officers, psychologists, nurses, and counsellors. Responses explored workload, resource availability, multidisciplinary team (MDT) working, patient autonomy, family involvement, medication practices, psychotherapy access, stigma, risk management, admission duration, and discharge processes. Data were analysed inductively to identify recurring and contrasting themes across the two service models.

**Results:**

Six interrelated themes emerged.

First, patient volume and workload were markedly higher in government services, with clinicians reporting time-pressured assessments and limited follow-up, whereas private services had lower patient volumes but greater documentation and accountability requirements.

Second, resource availability differed substantially, with chronic shortages of staff, investigations, and therapeutic spaces in government settings compared to better infrastructure in private care.

Third, multidisciplinary team (MDT) working was informal or fragmented in government services but more structured and routine in private hospitals.

Fourth, patient autonomy and family involvement were constrained in government settings due to workload and system pressures, while private services allowed greater shared decision-making, though sometimes influenced by family finances.

Fifth, medication and psychotherapy practices showed guideline-based intentions in both sectors; however, polypharmacy and irregular follow-up were more common in government care, while psychotherapy access and monitoring were more consistent privately.

Finally, risk management, admission duration, and discharge planning were reactive and bed-pressure driven in government services, compared with more planned and individualised pathways in private settings.

**Conclusion:**

The findings support the hypothesis that government and private psychiatric services in Bangladesh offer contrasting but complementary strengths. Government services provide accessibility and broad clinical exposure, while private services offer structure, continuity, and multidisciplinary care. Hybrid service models integrating these strengths may improve quality, equity, and sustainability of mental healthcare in similar low- and middle-income countries.

